# Role of telomere maintenance genes as a predictive biomarker for colorectal cancer immunotherapy response and prognosis

**DOI:** 10.17305/bb.2025.12053

**Published:** 2025-07-02

**Authors:** Zhikai Wang, Chunyan Zhao, Yifen Huang, Chong Li

**Affiliations:** 1 Department of Gastrointestinal Surgery, Henan Provincial People’s Hospital, Zhengzhou, China; 2 Department of Oncology, The Affiliated Dazu’s Hospital of Chongqing Medical University, Chongqing, China; 3 Outpatient Department, The Affiliated Dazu’s Hospital of Chongqing Medical University, Chongqing, China

**Keywords:** Immunotherapy, risk model, prognostic signature, telomere maintenance gene, TMG, colorectal cancer, CRC

## Abstract

Colorectal cancer (CRC) represents a significant global health challenge. Although telomere maintenance plays a crucial role in tumorigenesis, the prognostic value and immunotherapeutic relevance of telomere maintenance genes (TMGs) in CRC remain poorly understood. In this study, relevant data were retrieved from The Cancer Genome Atlas (TCGA) and the Gene Expression Omnibus (GEO) databases. TMG scores were calculated using the single-sample gene set enrichment analysis (ssGSEA) method, and TMGs associated with prognosis were subsequently identified. TCGA-CRC samples were classified into subtypes via consensus clustering (ConsensusClusterPlus). A risk prediction model was then constructed using univariate and Lasso Cox regression analyses. Survival analysis was performed using Kaplan–Meier curves generated with the survival package. Key genes were validated *in vitro* using cellular models. Immune cell infiltration was evaluated through ssGSEA, TIMER, and MCP-Counter tools, and chemotherapy responses were predicted using the pRRophetic package. Based on the results, 28 prognosis-related TMGs, two distinct CRC subtypes were established, with subtype C1 demonstrating more favorable clinical outcomes. Additionally, a risk model incorporating seven TMG-related genes (CDC25C, CXCL1, RTL8C, FABP4, ITLN1, MUC12, and ERI1) was developed for CRC prognosis. Differential mRNA expression levels of these genes were confirmed between CRC cell lines and normal control cells. Furthermore, silencing MUC12 suppressed CRC cell migration and invasion *in vitro.* Importantly, CRC patients classified as low-risk exhibited superior responses to immunotherapy, whereas high-risk patients showed increased sensitivity to conventional anti-cancer treatments. This study represents the first systematic evaluation of TMGs in CRC prognosis and immunotherapy, providing novel insights that could inform personalized therapeutic strategies.

## Introduction

Colorectal cancer (CRC) ranks among the most prevalent malignancies of the digestive system [1, 2], with projections suggesting it will affect approximately 2.5 million individuals by 2035 [3]. Globally, CRC accounts for 9.6% of all cancer cases, making it the third most frequently diagnosed cancer [4]. It is also the second leading cause of cancer-related death, responsible for 9.3% of deaths, which translates to approximately 900,000 deaths worldwide each year [4, 5]. Due to its asymptomatic nature in the early stages, timely diagnosis and treatment remain challenging, underscoring the need for more advanced screening methods [6, 7]. While studies have identified transcription factors (e.g., Nrf2) and ferroptosis-related genes (such as GSH, GPX4, and P53) as potential therapeutic targets for CRC [8], the lack of reliable biomarkers continues to hinder the accuracy of prognostic predictions [9]. Therefore, identifying biomarkers specific to CRC and developing robust prognostic models are crucial for the early detection and prognosis of the disease [10].

Telomeres, which consist of protective proteins and TTAGGG repeats [11, 12], are nucleoprotein complexes located at the ends of human chromosomes. Studies have shown that telomeres play essential roles in maintaining chromosomal and genomic stability [13]. However, during certain cell divisions or disease states, telomere length gradually shortens [14, 15]. To maintain their length, telomerase adds TTAGGG sequences to the chromosomal ends [16, 17]. Increasing evidence links the shortening of telomere length to the development of various diseases, including tumors [13, 18, 19]. It has been observed that telomere maintenance genes (TMGs) influence cancer occurrence by regulating mutations in the telomerase reverse transcriptase (TERT) promoter, suggesting their potential as cancer biomarkers [20, 21]. Two mechanisms for telomere elongation [22] have been identified in cancers: telomerase activation [23] and alternative lengthening of telomeres (ALT) [24]. Telomerase overexpression is commonly observed in many tumor cells [25, 26], while abnormal activation of ALT has been detected in telomerase-negative tumors, such as those in thyroid cancer [27]. Despite these findings, the prognostic value of TMGs in CRC remains unclear and warrants further investigation to enhance current therapeutic strategies.

This study was designed to systematically evaluate the expression patterns, molecular subtypes, and prognostic significance of TMGs in CRC, along with their interactions with the immune microenvironment and correlations with treatment responses. Using transcriptomic data from public CRC databases, the single-sample gene set enrichment analysis (ssGSEA) method was employed to compute TMG expression scores, and key survival-related genes were identified. Molecular subtypes were classified based on prognostically correlated TMGs using a consensus clustering method. Differential expression and functional enrichment analyses were then performed to explore the potential biological mechanisms. A RiskScore model was developed to assess the performance of TMGs in evaluating immune cell infiltration, immunotherapy response, chemotherapy sensitivity, and survival outcomes in CRC patients. Finally, *in vitro* experiments were conducted to validate the expression and biological functions of key genes. Collectively, our findings highlight the critical role of TMGs in immune regulation and CRC development, providing new insights for personalized treatment and risk assessment.

## Materials and methods

### Acquisition and preprocessing of data

CRC data, including clinical information for both colon and rectum adenocarcinomas, copy number variations (CNVs), and somatic mutations, were retrieved from The Cancer Genome Atlas (TCGA) database (https://portal.gdc.cancer.gov/) and termed as “TCGA-CRC” cohort. After excluding samples without survival data, 589 tumor samples with survival times greater than 0 days were retained. The RNA-seq expression profiles were converted to TPM format and log2-transformed. Additionally, we downloaded GSE17537 microarray data from Gene Expression Omnibus (GEO, https://www.ncbi.nlm.nih.gov/geo) and converted the probes to gene symbols based on the annotation file. After excluding samples without clinical follow-up or survival data, 55 tumor samples from GSE17537 were retained. Finally, 2093 TMGs were extracted from a previous study [28].

### Identification of CRC-related TMGs and analysis of their mutations and CNVs

Using the ssGSEA method, TMG scores for the TCGA-CRC dataset were computed. DEGs between tumor and adjacent tissues were identified and intersected with TMG signatures. Prognosis-correlated TMGs were then selected through univariate Cox regression analysis. CNV and mutation data from the TCGA database were collected. Briefly, MuTect2 [29] was used to analyze the mutational landscape of TMG in primary CRC samples from the TCGA-CRC cohort, followed by visualization of the mutational status in a waterfall plot with the maftools R package [30]. The CNV status of the TMGs in primary CRC samples was then assessed using ADTEx [31].

### Molecular clustering

Consensus clustering was performed on the tumor samples using the ConsensusClusterPlus package [32], with hierarchical clustering (clusterAlg ═ “hc”) and Pearson correlation distance (distance ═ “pearson”) as the clustering parameters. The analysis was conducted over 500 iterations with a resampling rate of 80%. The optimal number of clusters (*k*) was determined based on the stabilized cumulative distribution function (CDF) curve, minimal incremental gains in the delta area plots, and high intra-cluster consensus with clear inter-cluster separation in the consensus matrices. Finally, the clinical features (M.stage, N.stage, T.stage, stage, status, age, and gender) and prognosis across the different molecular subtypes were systematically assessed.

### Enrichment analysis

DEGs between C1 and C2 were identified using the limma package (false discovery rate < 0.05 & |log2FC| >log2(1.5)) [33] to select common genes for enrichment analysis. The TCGA-CRC cohort was then subdivided into two molecular subtypes based on the DEGs. Next, the clusterProfiler R package [34] was used to conduct GO and KEGG enrichment analysis [35].

### Establishment of a risk model

The prognosis-correlated DEGs were screened using univariate Cox regression analysis, followed by refinement of the risk model with Lasso Cox regression from the glmnet package [36] and 10-fold cross-validation. A RiskScore model was then formulated through stepwise multivariate Cox regression analysis as follows: (1)
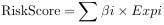


Where βi represents the coefficient of a gene in the Cox regression model, and Expi represents the gene expression. The samples were divided into low- and high-risk groups based on the median RiskScore as the threshold. Survival differences between the two groups were then analyzed using Kaplan–Meier (KM) curves with the survival package [37]. The prognostic classification of the RiskScore model was validated through receiver operating characteristic (ROC) analysis using the timeROC R package [38] and principal component analysis (PCA). Additionally, the prognostic differences between the two risk groups across gender, age, and stage were compared by calculating the RiskScores for all patients. Independent clinical and pathological factors (stage, M.stage, N.stage, T.stage, status, age, and gender) for CRC prognosis were selected using univariate and multivariate Cox regression analyses, along with the RiskScore model. To predict 1-, 3-, and 5-year survival for CRC patients, a prognostic nomogram was developed using the rms package [39]. Finally, the clinical reliability of the nomogram was assessed by calibration curve analysis and decision curve analysis (DCA).

### Tumor microenvironment (TME) differences across the risk groups

Immune cell infiltration in each risk groups was comprehensively examined using the ssGSEA algorithm, the MCPcounter package [40], and the TIMER online tool (http://cistrome.org/TIMER). Specifically, the TIMER offer six main analysis modules to enable the correlation analysis between a range of factors and immune infiltration.

### Culture and transfection of cells

Human colon adenocarcinoma cells (SW1116, BNCC100262) and normal human colon epithelial cells (NCM460, BNCC339288), both obtained from the BeiNa Culture Collection (Xinyang, China), were cultured in either RPMI Medium 1640 (31800, Solarbio Lifesciences, Beijing, China) or Leibovitz’s L-15 Cell Culture Medium (LA9510, Solarbio Lifesciences). Both media were supplemented with 10% FBS (S9020, Solarbio Lifesciences). The cultures were maintained at 37 ^∘^C to ensure optimal growth conditions. For NCM460 cells, the culture was performed in an incubator with 5% CO_2_. The siRNA was synthesized by GenePharma (Suzhou, China) and transfected into SW1116 cells using Lipofectamine 2000 (11668027, Invitrogen, Carlsbad, CA, USA). The negative control, which contained a scrambled target sequence (5′-ACCAGTATTGGAGGTAATACAAC-3′), was purchased [41].

### Wound healing assay

A controlled artificial wound was created on the cell monolayers using a 10 µL pipette tip after culturing the transfected CRC cells SW1116 (1 × 10^5^ cells/well) to full confluence in a 6-well plate. The cells were then cultured in serum-free medium at 37 ^∘^C. Photographs were taken at 0 hour and 48 hours (h) using an Eclipse Ts2R microscope (Nikon, Tokyo, Japan).

### Transwell assay

Matrigel (50 µL, Corning, Inc., Corning, NY, USA) was used to pre-coat the transwell chambers (pore size: 8 µm, 3422, Corning, Inc.), which were placed on 24-well plates. Next, the transfected SW1116 cells in serum-free medium (200 µL) were planted into the upper chamber at 1 × 10^5^ cells/well, whereas the lower transwell chamber contained 700 µL complete medium. After cell culture for 48 h, the cells were fixed by 4% paraformaldehyde (P1110, Solarbio Lifesciences) for 30 minutes (min), followed by using 0.1% crystal violet solution (G1063, Solarbio Lifesciences) for cell staining another 15 min. Finally, the cells were observed and quantified under the microscope used in the previous assay.

### QRT-PCR analysis

Total RNA was extracted from NCM460 and SW1116 cells using the TriZol kit (15596026CN, Invitrogen, Carlsbad, CA, USA) and then reverse transcribed into cDNA with a first-strand cDNA synthesis kit (1708890, Bio-Rad Laboratories, Hercules, CA, USA). Quantitative PCR (qPCR) amplification was performed using a CFX384 qPCR System (1855484, Bio-Rad Laboratories) with SYBR Green Supermix (1708880, Bio-Rad Laboratories), under the following cycling conditions: initial denaturation at 95 ^∘^C for 2 min, followed by 40 cycles of denaturation at 95 ^∘^C for 15 s, annealing at 60 ^∘^C for 30 s, and extension at 72 ^∘^C for 30 s. GAPDH was used as the normalization control, and relative mRNA expression was calculated using the 2^‑ΔΔct^ method [42]. Primers are listed in Table S1.

### Immunotherapy and drug sensitivity

Immunotherapy responses were predicted by standardizing the transcriptome data applying TIDE (http://tide.dfci.harvard.edu/) to calculate the TIDE scores, with higher TIDE scores showing greater possibility of immune escape and less immunotherapy benefit. Next, chemotherapy sensitivity in the TCGA-CRC dataset and the differences of patients’ responses were analyzed and compared by the pRRophetic software package [43]. Patients’ sensitivity in different risk groups to chemotherapy agents was evaluated with IC_50_.

### Statistical analysis

All computational analyses were conducted using R (version 3.6.0). The normality of data distribution was assessed with the Shapiro–Wilk test prior to testing for variance. For comparisons between two independent groups of continuous variables, the Wilcoxon rank-sum test was applied. The Kruskal–Wallis test was used to examine differences in continuous variables among three groups. The chi-square test was employed to assess disparities in categorical variables across different groups. Additionally, the log-rank test was used to compare survival times between patients in different groups. *P* < 0.05 denoted a statistical significance. For *in vitro* cellular experiments, differences between the normal and experimental groups were analyzed using Student’s *t*-test with GraphPad Prism software (version 8.0.2). Data are presented as the mean ± standard deviation (SD). Analytical support for this study was provided by SangerBox (http://sangerbox.com/) [44].

## Results

### Genomic landscape of the TMGs in CRC

**Figure 1.**
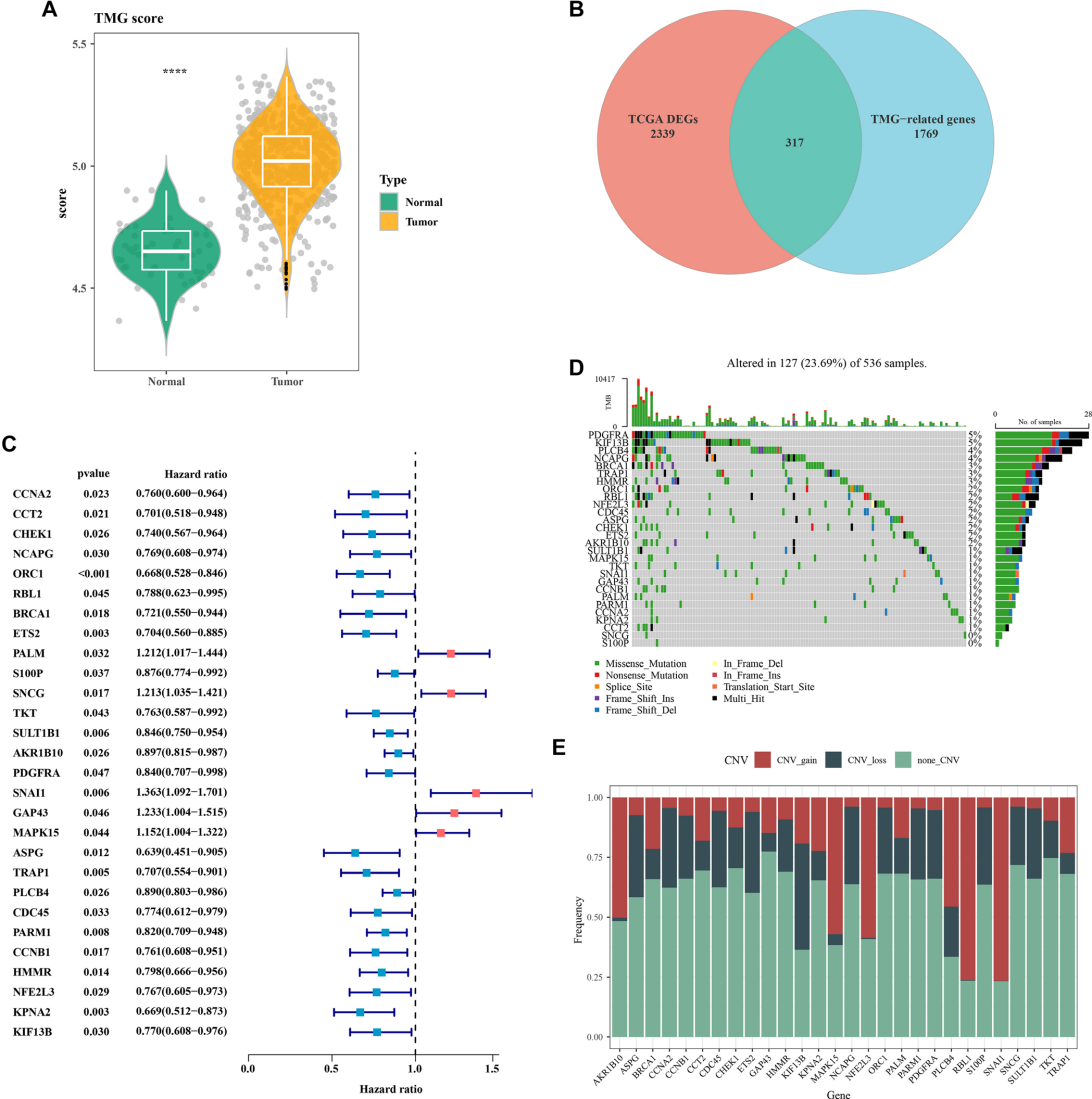
**Genomic landscape analysis of TMGs in CRC.** (A) Expression of TMG scores in CRC and adjacent non-cancerous control samples; (B) Differential genes from tumor and adjacent tissues intersected with TMG; (C) Identification of 28 TMGs closely associated with CRC prognosis; (D) Mutation status of TMGs in CRC; (E) CNVs of TMGs in CRC. ****P < 0.0001. CRC: Colorectal cancer; TMG: Telomere maintenance genes; CNVs: Copy number variations.

Using ssGSEA analysis, we first computed the TMG scores in the TCGA-CRC dataset. The results showed that tumor tissues exhibited significantly higher TMG scores than adjacent non-tumor tissues (Figure 1A). Further analysis identified 317 DEGs between the two tissue types (Figure 1B), among which 28 TMGs were found to have significant prognostic relevance in CRC based on univariate Cox regression analysis (Figure 1C). Additionally, an analysis of the mutational status and CNVs of these 28 TMGs in the tumor samples revealed that only 23.69% of the samples carried gene mutations associated with telomere maintenance (Figure 1D). Among these, SNAI1 and RBL1 showed relatively higher frequencies of copy number amplification (Figure 1E).

### Identification of CRC molecular subtypes based on TMGs

**Figure 2.**
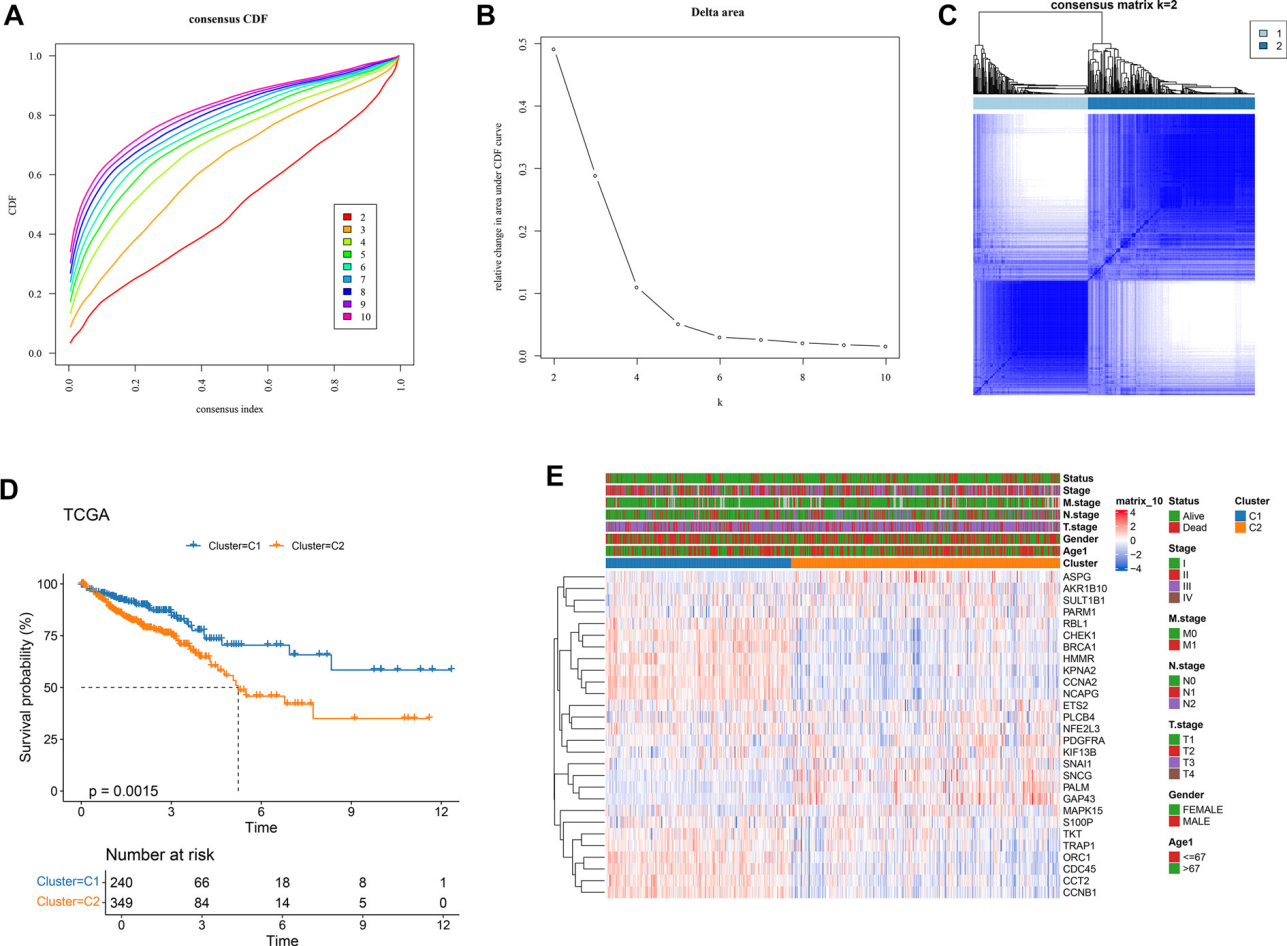
**Classification and prognostic variations amongTCGA-CRC samples.** (A) CDF curve plotted for TCGA-CRC cohort samples; (B) CDF delta area curve generated for the TCGA-CRC cohort; (C) Heatmap of sample clustering at consensus *k* ═ 2 in the TCGA-CRC cohort; (D) KM curve illustrating the relationship between OS and two subtypes in the TCGA-CRC cohort; (E) Heatmaps of clinical features (status, stage, M stage, N stage, T stage, age, and gender) and expression levels between subtypes in the TCGA-CRC cohort. TCGA: The Cancer Genome Atlas; CRC: Colorectal cancer; CDF: Cumulative distribution function; KM: Kaplan–Meier; OS: Overall survival.

In this study, we use consistency clustering to analyze TCGA-CRC samples, utilizing a combination of CDF curves, Delta Area plots, and consistency matrix heatmaps. The stability and reasonableness of different clustering numbers (*k*) are evaluated by examining these plots. First, the CDF curves (Figure 2A) show that as the number of clusters increases from *k* ═ 2 to *k* ═ 3, the curves shift toward the upper right corner, indicating improved clustering consistency. However, the improvement is significantly smaller after *k* ═ 3. Figure 2B further demonstrates that the largest increment in CDF area occurs between *k* ═ 2 and *k* ═ 3, with diminishing returns at *k* ≥ 4, suggesting limited benefits to increasing the number of clusters beyond *k* ═ 3. Additionally, Figure 2C shows that with *k* ═ 2, the heatmap reveals high consistency within clusters, clear separation between clusters, and a robust clustering structure. Based on these analyses, we determined that *k* ═ 2 is the optimal number of clusters, dividing the samples into two groups: C1 and C2. Survival analysis indicated significantly better overall survival (OS) in C1 compared to C2 (Figure 2D, *P* ═ 0.0015). Clinical feature analysis revealed differences in M.stage and status between the two subtypes. Comparison of TMG expression profiles showed that C1 had higher expression levels of multiple genes, including RBL1, CHEK1, BRCA1, HMMR, KPNA2, CCNA2, NCAPG, TKT, TRAP1, ORC1, CDC45, CCT2, and CCNB1 (Figure 2E). These findings confirm the robust classification of CRC samples into two molecular subtypes with significant survival differences and clinical heterogeneity, providing a strong foundation for future molecular subtyping studies.

### Enrichment analysis results of the DEGs

**Figure 3.**
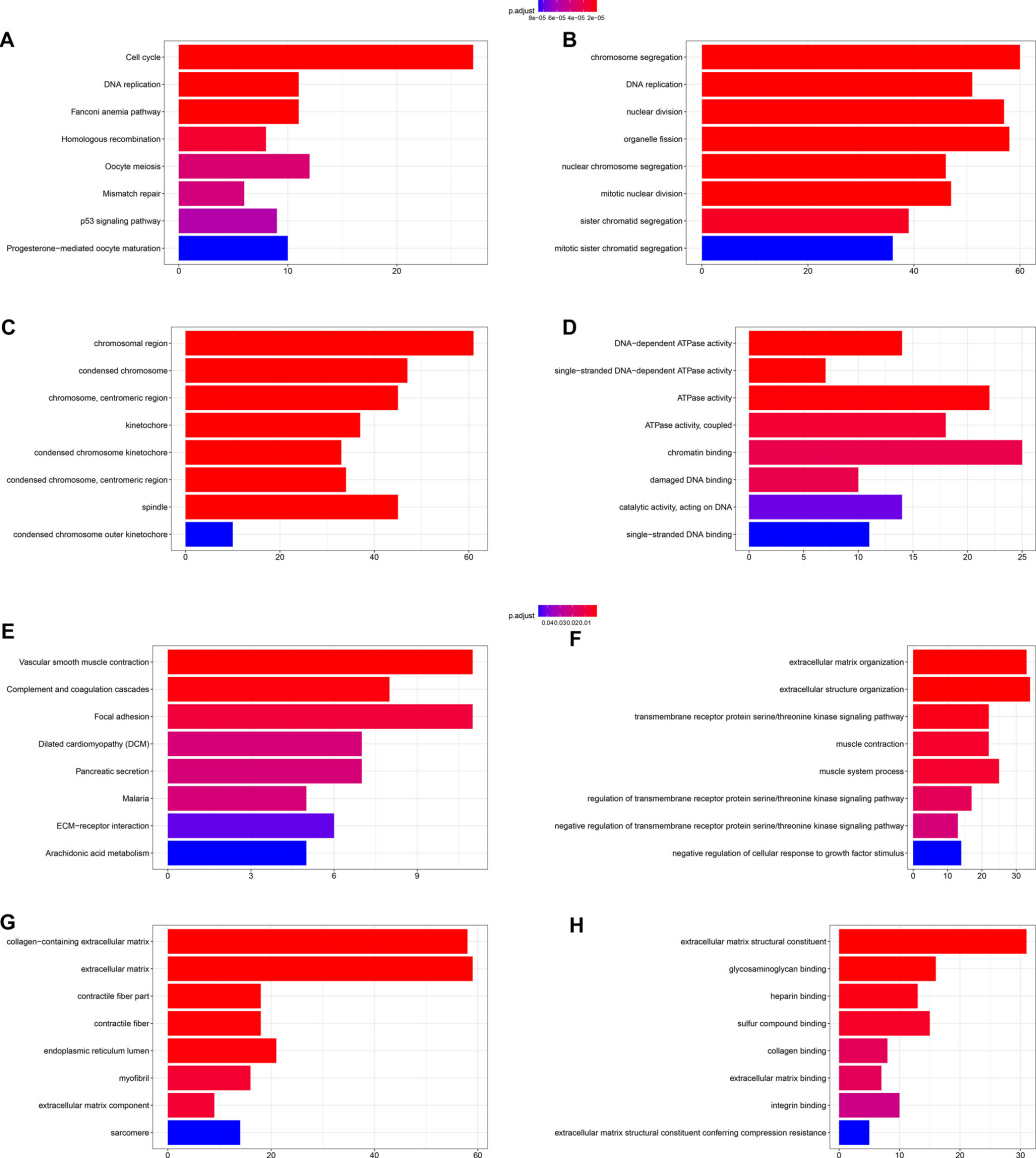
**Differential gene identification and enrichment analysis.** (A–D) DEGs enrichment analysis for the C1 subtype in the TCGA-CRC cohort; (E–H) DEGs enrichment analysis for the C2 subtype in the TCGA-CRC cohort. DEGs: Differentially expressed genes; TCGA: The Cancer Genome Atlas; CRC: Colorectal cancer.

Differential expression analysis using limma package [45] identified 538 DEGs between C1 and C2 (282 upregulated in C1, 256 upregulated in C2). GO and KEGG enrichment analysis showed that C1-associated genes were mainly enriched in proliferation-related pathways including DNA replication and cell cycle (Figure 3A–3D), while C2-associated genes were mainly enriched in pathways related to cancer metastasis and invasion such as focal adhesion, extracellular matrix (ECM) organization, ECM–receptor interaction (Figure 3E–3H). These results demonstrated significant differences between the two subtypes in terms of potential therapeutic response and biological behaviors in CRC.

### Development of a risk model based using the seven TMGs

**Figure 4.**
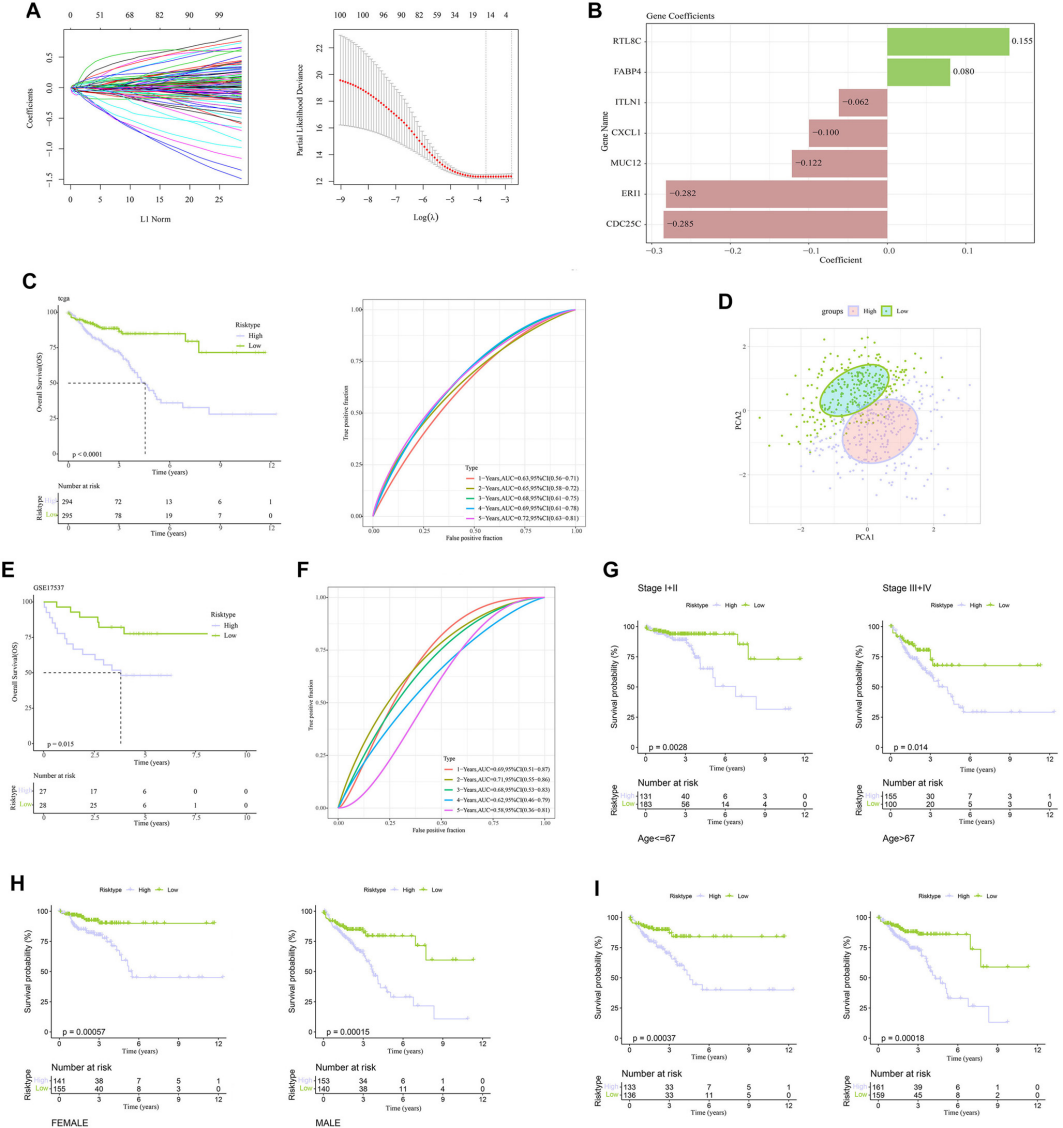
**Development and validation of a TMGs-based risk model.** (A) LASSO Cox regression analysis performed to evaluate DEGs associated with CRC prognosis in the TCGA-CRC training cohort; (B) Risk coefficients of key genes in the TCGA-CRC training cohort; (C) KM survival curve and ROC curve for 1-, 3-, and 5-year prognostic predictions for the TCGA-CRC training cohort; (D) PCA comparing low- and high-risk groups within the TCGA-CRC training cohort; (E and F) KM survival curves and ROC curves for the model based on the GEO testing dataset; (G–I) Prognostic differences between the two risk groups across various tumor stages (G), ages (H), and genders (I). DEGs: Differentially expressed genes; CRC: Colorectal cancer; TCGA: The Cancer Genome Atlas; KM: Kaplan–Meier; ROC: Receiver operating characteristic; PCA: Principal component analysis; TMGs: Telomere maintenance genes.

Through univariate Cox analysis, we identified 101 prognostic DEGs (*P* < 0.05), which were finally refined to seven key genes (*CDC25C, CXCL1, RTL8C, FABP4, ITLN1, MUC12,* and *ERI1*) by Lasso Cox regression analysis with 10-fold cross-validation (Figure 4A) and stepwise multivariate Cox regression analysis (Figure 4B). The formula of the RiskScore model was as follows: (1)
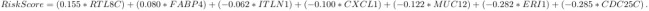


**Figure 5.**
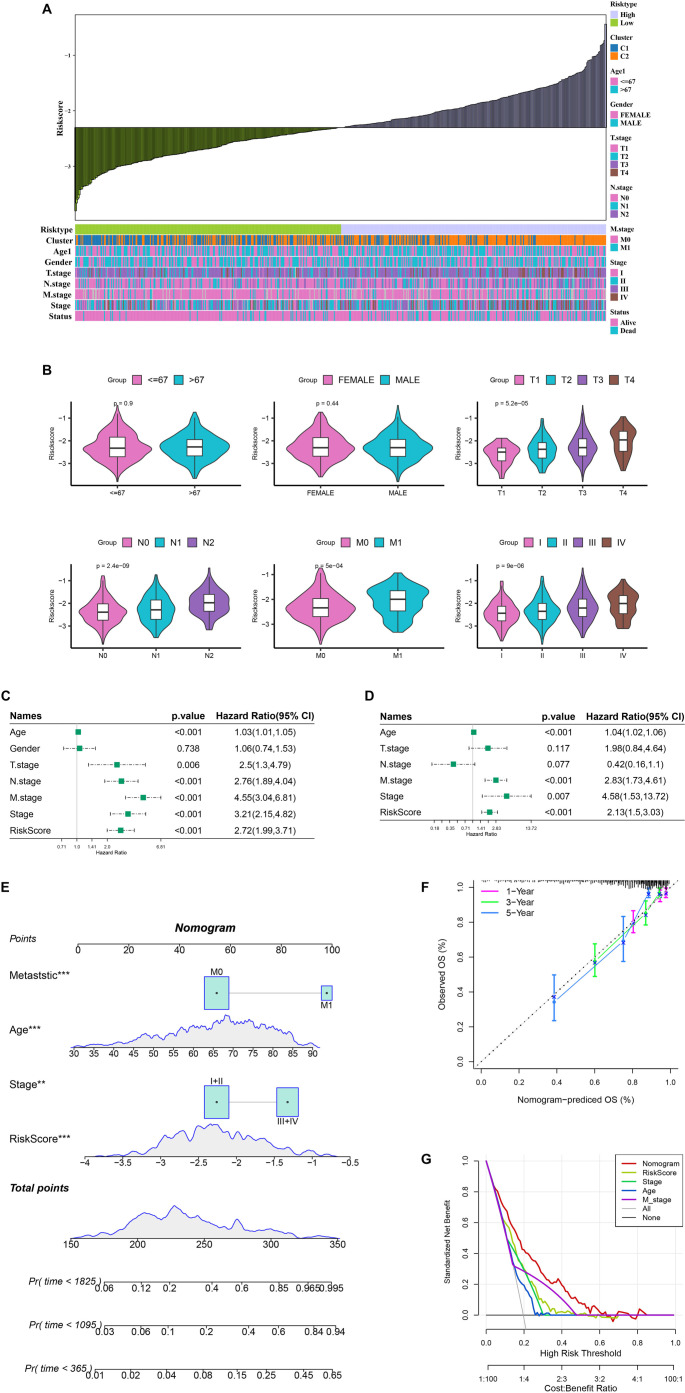
**Nomogram development for CRC prognosis assessment.** (A) Relationship between RiskScore and clinical characteristics (M stage, N stage, T stage, overall stage, status, age, and gender); (B) Violin plots illustrating the distribution of clinical characteristics (status, M stage, N stage, T stage, overall stage, age, and gender) between low-risk and high-risk groups; (C and D) Univariate and multivariate Cox regression analyses conducted to assess the impact of RiskScore and clinical characteristics (status, stage, M stage, N stage, T stage, age, and gender); (E) Nomogram to predict 1-, 3-, and 5-year OS for CRC patients; (F) Calibration curve validating the established nomogram; (G) DCA of the nomogram. **P < 0.01; ***P < 0.001. CRC: Colorectal cancer; DCA: Decision curve analysis; OS: Overall survival.

Patients were allocated by the median value of RiskScore into low-risk and high-risk groups. KM survival curve demonstrated that the two risk groups in the TCGA-CRC training cohort differed significantly in patients’ survival, with those having a higher RiskScore showing shorter OS (Figure 4C). The timeROC package [38] was employed in ROC analysis for further validating the prognosis classification of the RiskScore. The AUC values in the training dataset for 1-, 3-, and 5-year survival were 0.63, 0.68, and 0.72, respectively, which suggested a highly accurate survival evaluation by the model (Figure 4C). Furthermore, PCA results also showed a distinct separation between the two risk groups in the TCGA-CRC cohort (Figure 4D), further supporting the performance of the RiskScore in identifying CRC patients with different risks.

The robustness of the RiskScore was confirmed using the validation dataset GSE17537 (Figure 4E and 4F). Consistently, the RiskScore value showed a negative correlation with survival outcomes in this dataset. A comparison of the performance between the low- and high-risk groups across different clinical factor subgroups revealed significant differences (*P* < 0.05), independent of stage classification (I + II vs. III + IV), gender (male vs. female), or age (> 67 or ≤ 67). This suggests that the RiskScore provides an independent risk classification, less likely to be influenced by other clinical factors (Figure 4G–4I).

### Validation of the TMG-related risk model

Analysis of the TCGA-CRC cohort revealed strong associations between risk groups and pathological staging, with the high-risk group showing a higher prevalence of the C2 subtype and metastasis cases. This finding aligns with our previous research, which demonstrated that the C2 cluster is enriched in metastasis-related pathways. Further analysis indicated that the RiskScore correlated positively with more advanced clinical stages (T.stage, N.stage, and overall stage). Figure 5A and 5B illustrates the relationship between clinical features and RiskScore, along with a violin plot, respectively. Both univariate and multivariate Cox regression analyses identified M.stage, stage, RiskScore, and age as significant prognostic factors for CRC (Figure 5C and 5D). A nomogram was developed by integrating other clinical and pathological characteristics with the RiskScore to estimate survival and risk for CRC patients (Figure 5E). The results highlighted the RiskScore as the most influential factor for survival prediction. Calibration curves revealed that the 1-, 3-, and 5-year prediction curves closely aligned with the reference curve (Figure 5F), indicating strong predictive performance of the nomogram. Additionally, DCA demonstrated superior clinical effectiveness and reliability of the nomogram (Figure 5G).

### *In vitro* verification of the key genes for CRC prognosis

**Figure 6.**
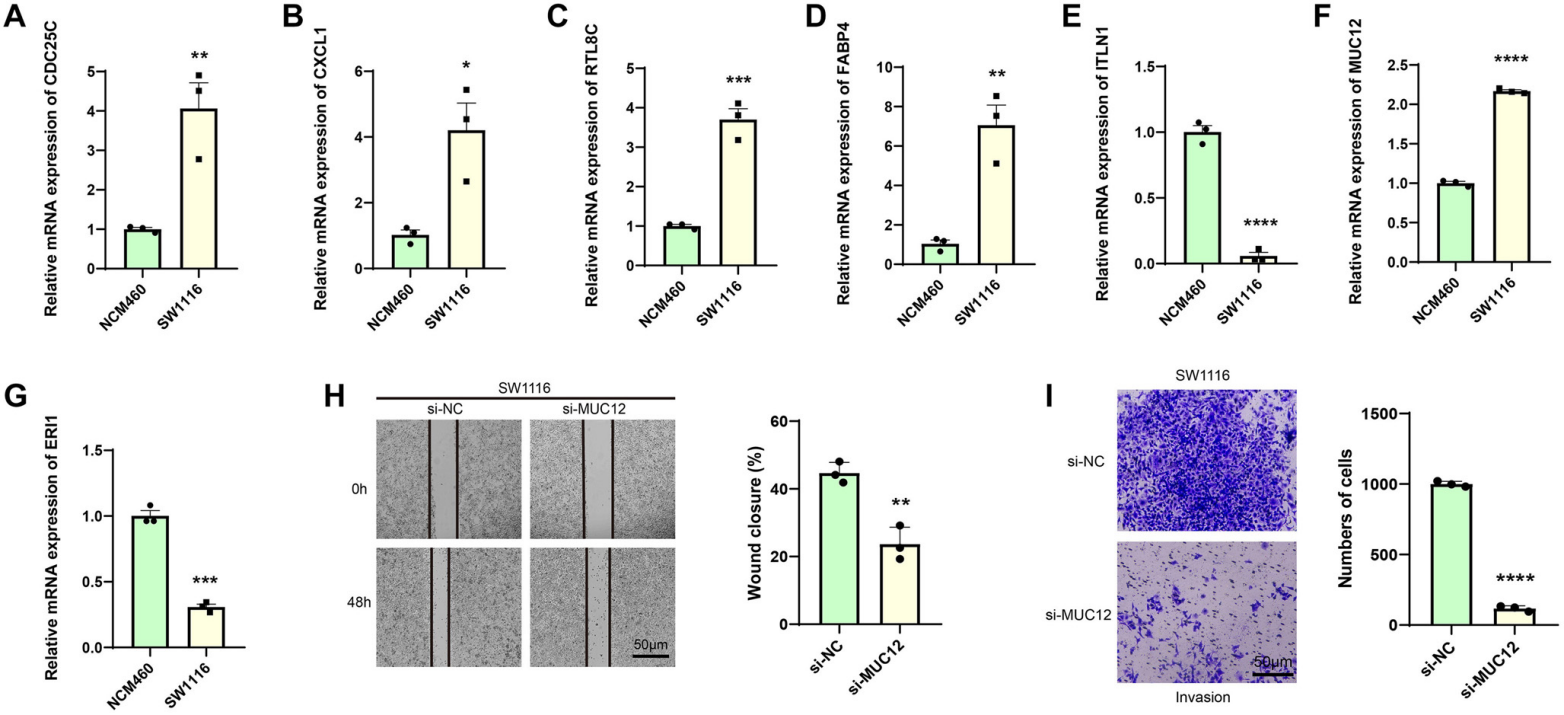
***In vitro* validation using CRC cells.** (A–G) Quantified expression levels of seven biomarkers: CDC25C (A), CXCL1 (B), RTL8C (C), FABP4 (D), ITLN1 (E), MUC12 (F), and ERI1 (G) in CRC cells (SW1116) and human normal colonic epithelial cells (NCM460) via qRT-PCR. (H) Impact of MUC12 silencing on the migration of CRC cells SW1116 assessed through a wound healing assay. (I) Effects of MUC12 silencing on the invasion of CRC cells SW1116 evaluated via a transwell assay. Data from three independent experimental sets are presented as mean ± standard deviation. *P < 0.05; **P < 0.01; ***P < 0.001; ****P < 0.0001. CRC: Colorectal cancer; qRT-PCR: Real-time quantitative reverse transcription PCR.

The relative expressions of the seven genes (*CDC25C*, *CXCL1*, *RTL8C*, *FABP4*, *ITLN1*, *MUC12*, and *ERI1*) in SW1116 and NCM460 cells were measured. It was found that the expressions of *ITLN1* and *ERI1* were notably downregulated in SW1116 cells, while the mRNA expressions of *CDC25C*, *CXCL1*, *RTL8C*, *FABP4*, and *MUC12* were significantly higher in SW1116 cells than in control NCM460 cells (Figure 6A–6G, *P* < 0.05).

Previous study found the potential of *MUC12* as a molecular marker for the prognosis of CRC [46, 47]. Therefore, this study performed wound healing and transwell assays to evaluate the potential effects of *MUC12* knockdown on CRC cells. As shown in Figure 6H and 6I, *MUC12* knockdown notably suppressed the migration and invasion abilities of SW1116 cells (*P* < 0.01). This result was consistent with the cancer-promoting role of *MUC12*, which further supported the clinical significance of the RiskScore model developed based on TMGs.

### Differences in the TME between CRC patients with different risks

**Figure 7.**
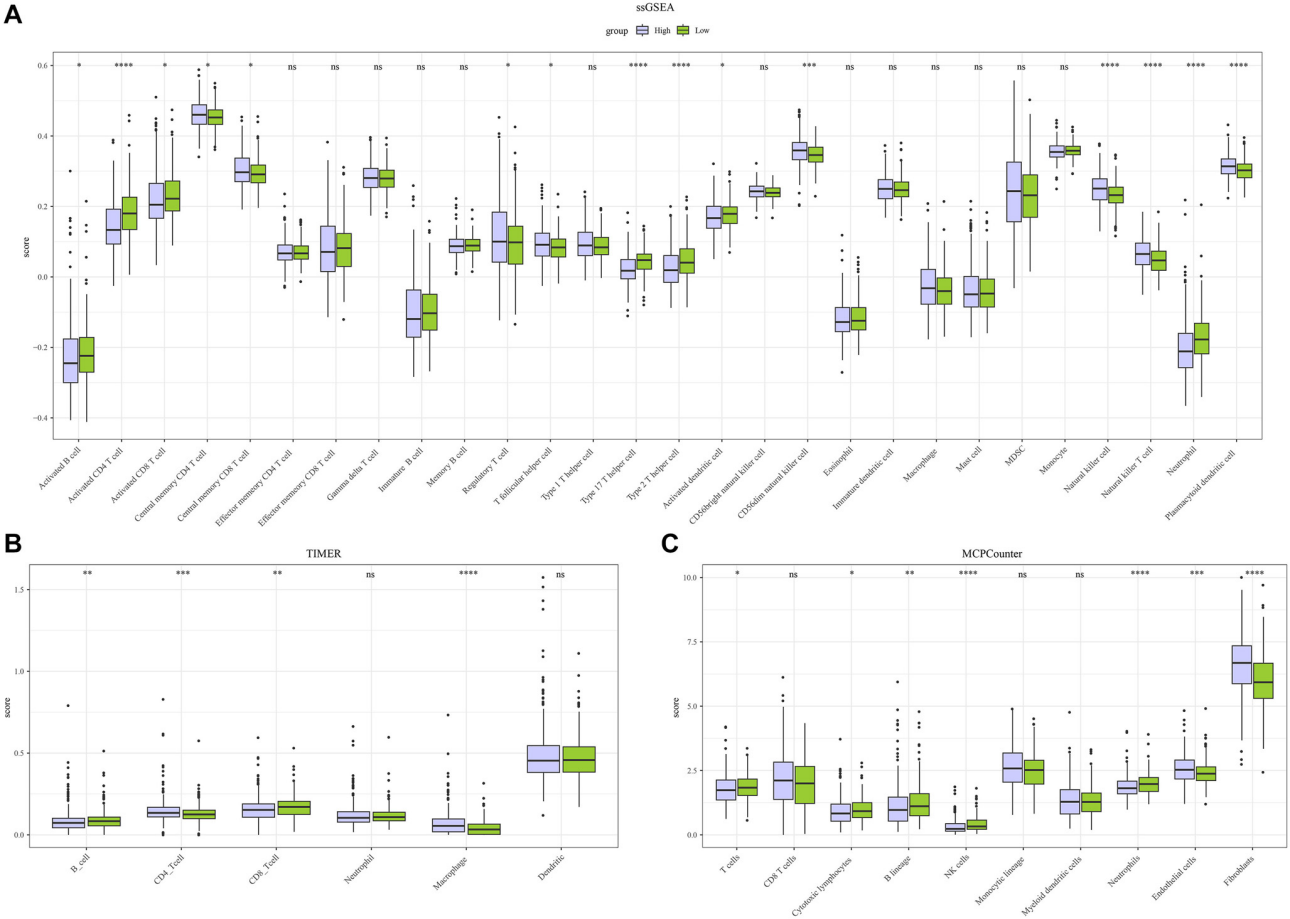
**Analysis of immune infiltration levels between high- and low-risk groups.** (A–C) Immune infiltration levels for the two risk groups were calculated using (A) ssGSEA, (B) TIMER, and (C) MCPcounter methods, respectively. ^ns^*P* < 0.05; *P < 0.05; **P < 0.01; ***P < 0.001; ****P < 0.0001.

The ssGSEA analysis showed that the infiltration of Type 17 T helper cells, neutrophils, activated B cells, Type 2 T helper cells, activated CD4 T cells, and activated CD8 T cells—immune cells typically involved in killing tumor cells [48]—was lower in the high-risk group compared to the low-risk group. This reduced infiltration may contribute to the protection of tumor cells. Additionally, regulatory T cells, which are highly expressed in the high-risk group, could promote tumor development (Figure 7A). TIMER analysis further revealed that CD8+ T cells and B cells were less expressed in the high-risk group (Figure 7B). MCPcounter analysis (Figure 7C) identified significant differences in the infiltration of several cell types between the two groups. Specifically, the high-risk group exhibited significantly lower infiltration of NK cells, cytotoxic lymphocytes, T cells, neutrophils, and B lineage cells. These findings suggest that the absence of immune effector cells may contribute to a “cold-immune” TME in high-risk patients, which could explain their poorer prognosis.

### Immunotherapy and drug sensitivity analysis for CRC patients in different risk groups

**Figure 8.**
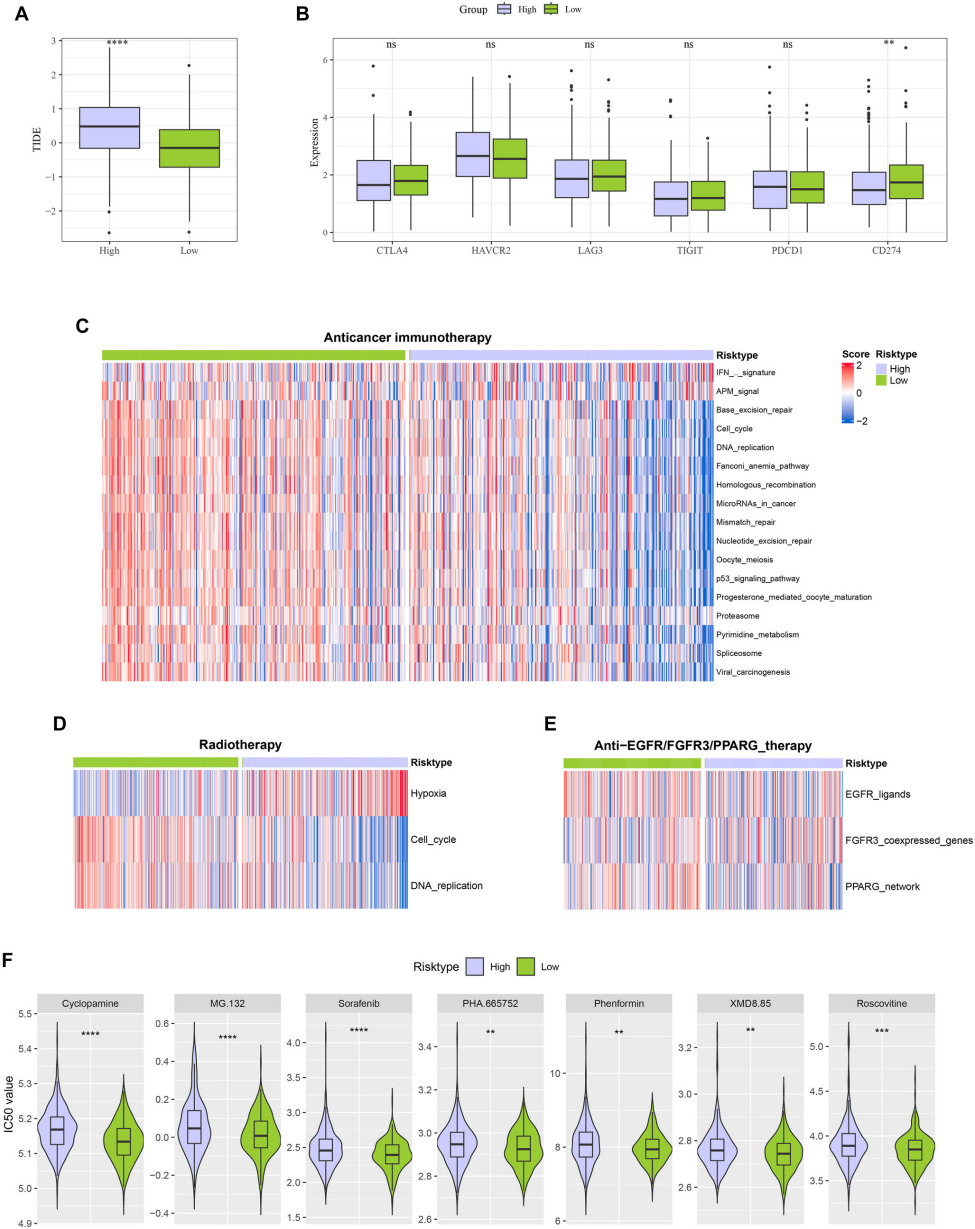
**Immunotherapy and drug sensitivity studies between high- and low-risk groups.** (A) Differences in TIDE scores between high-risk and low-risk groups; (B) Variations in common immune checkpoint expressions between high-risk and low-risk groups; (C–E) Responses to (C) anticancer immunotherapy, (D) radiotherapy, and (E) anti-EGFR/FGFR3/PPARG therapy between high-risk and low-risk groups; (F) Differences in drug sensitivity to Cyclopamine, MG-132, Sorafenib, PHA-665752, Phenformin, XMD8.85, and Roscovitine observed between high-risk and low-risk groups. ^ns^*P* < 0.05; **P < 0.01; ***P < 0.001; ****P < 0.0001.

TIDE analysis revealed significantly lower TIDE scores in the low-risk group (Figure 8A), suggesting more active immune response and less immune evasion possibility in those patients. Further analysis showed that the low-risk group had a significantly higher expression level of the immune checkpoint inhibitor CD274 (PD-L1) than the high-risk group (Figure 8B), indicating a better response of low-risk patients to immune checkpoint blockade therapy. Based on the ssGSEA algorithm and an established gene signature [49], the responsiveness to treatments including anticancer immunotherapy and chemotherapy was analyzed. It was observed that low-risk CRC patients had a higher reactivity across multiple treatment-correlated gene sets (Figure 8C–8E), while high-risk CRC patients had higher sensitivities to common anti-cancer drugs, for instance, Phenformin, MG.132, Cyclopamine, and Sorafenib (Figure 8F). These findings highlighted that patients in different risk groups responded differently to the therapeutic strategies, with low-risk patients benefiting more from immunotherapy and high-risk patients benefiting more from conventional anti-tumor drug treatments.

## Discussion

CRC diagnosis remains challenging due to its asymptomatic nature in the early stages, which results in a poor prognosis. This underscores the need for effective prognostic biomarkers to reduce the mortality rate in CRC patients [7]. Telomere length in lymphocytes is closely linked to tumor development, and telomere shortening is considered a prognostic marker for CRC [50]. While genes like RCN3 have emerged as potential prognostic markers, their specific mechanisms still require further investigation [51]. Therefore, this study developed a novel TMG-based risk model for prognostic assessment in CRC to contribute to the field. Clustering analysis identified two distinct CRC subtypes (C1, C2) based on TCGA-CRC samples, with the C2 subtype exhibiting higher copy number and expression of SNAI1 and a poorer prognosis. SNAI1 has been found to play a pivotal role in maintaining telomere integrity [52], and its absence promotes telomerase activity in mesenchymal stem cells, highlighting the potential of SNAI1 as a crucial TMG in this process [52]. SNAI1 also regulates epithelial–mesenchymal transition (EMT) [53], a process during which epithelial cells lose their connections and polarity but acquire mesenchymal properties and invasive abilities [54]. Such phenotypic changes during EMT contribute to tumorigenesis. The expression level and function of SNAI1 have been widely studied in many types of cancer, including CRC. SNAI1 drives stem cell properties, metabolic alterations, cancer invasion, and chemoresistance in epithelial ovarian cancer [55], promotes metastasis in breast tumors [56], and high expression of SNAI1 is considered a clinical biomarker in gastric cancer [57]. In CRC, intestinal epithelial SNAI1 facilitates tumor development through EMT and the Wnt/β-catenin signaling pathway [58]. Furthermore, studies on both CRC patients and *in vitro* experiments have shown that SNAI1 expression predicts patient response to drug treatment [59]. This study found that SNAI1 had a higher CNV, consistent with previous findings that CNVs frequently occur in regions containing genes crucial for CRC, making them potential biomarkers for cancer detection [60]. Thus, this study proposed SNAI1 as a promising candidate for early CRC detection. Recent studies highlight the multifaceted roles of the seven identified TMGs (CDC25C, CXCL1, RTL8C, FABP4, ITLN1, MUC12, and ERI1) in carcinogenesis. For instance, CDC25C plays a critical role in regulating the G2/M phase of the cell cycle, and its expression changes are implicated in cancer growth [61]. CXCL1, a member of the CXC chemokine subfamily, demonstrates clinical significance in various cancer types [62]. FABP4, abundant in adipocytes, is upregulated in multiple solid tumors, indicating a poor prognosis [63]. RTL8C has potential as a promising pan-cancer biomarker [64]. High expression of ERI1 is linked to improved OS in CRC [65]. ITLN, primarily generated by stromal vascular fraction cells, plays a crucial role in cancer growth [66]. MUC12 is a type of transmembrane mucin typically expressed in the normal colon but less so in the pancreas. Studies have reported lower MUC12 mRNA levels in certain CRC tissues compared to normal colonic tissues [67, 68]. Notably, MUC12 exhibited functional complexity in this study. On one hand, multivariate Cox regression analysis revealed that MUC12 acts as an independent protective prognostic factor, with higher expression associated with longer OS. On the other hand, *in vitro* experiments showed that downregulation of MUC12 significantly suppressed CRC cell migration and invasion, suggesting a metastasis-promoting effect. The discrepancy between “statistically protective” and “functionally pro-carcinogenic” roles potentially indicates a dual function of MUC12 in different pathological stages or microenvironmental contexts. In early stages, MUC12 may play a protective role by maintaining epithelial barrier function, while its overexpression may contribute to EMT and microenvironmental remodeling, promoting tumor cell metastasis and invasion during tumor progression [69, 70]. These findings suggest that the specific role of MUC12 requires further elucidation through more *in vivo* mechanistic studies.

For cancer pathogenesis, the TME is a crucial factor, and its compositional changes can provide insights into patient responses to immunotherapy [71, 72]. In the present study, the high-risk CRC group showed a poorer prognostic outcome, which was consistent with its enrichment in the C2 cluster. We observed that the pathways enriched in C2 were primarily linked to cancer metastasis, suggesting that the worse prognosis in high-risk patients may be attributed to metastasis. High-risk patients also exhibited suppressed immune defense capabilities, leading to an upregulation of immune cell expression. In contrast, low-risk patients demonstrated a more robust immune response. Additionally, the high-risk group showed higher infiltration of endothelial cells and fibroblasts. Endothelial cells are key players in angiogenesis within cancerous tumors [59], a vital process that supplies oxygen and nutrients to tumors [73]. Fibroblasts, a type of mesenchymal cell, are involved in tissue homeostasis and disease processes [74]. The varying infiltration of different immune cell types within the TME may explain the distinctions between the two risk groups. The synergistic effects of fibroblasts and endothelial cells could contribute to tumor metastasis and spread, leading to a worse prognosis for high-risk CRC patients. Interestingly, high-risk patients exhibited an immune system that appeared “suppressed,” but they showed greater sensitivity to conventional anti-tumor agents. This suggests that in patients with significant immunosuppression, small molecule targeted therapies might be prioritized over immunotherapy alone.

There are several limitations in this study that should be acknowledged. First, while we utilized clinical data and large-scale RNA-seq data from public databases, potential biases could arise from inter-sample heterogeneity, differences in sequencing platforms, and incomplete clinical annotations. These factors may affect the generalizability of our model. Future multicenter prospective studies with larger, independent cohorts are needed to validate and enhance the clinical applicability and robustness of our risk model. Second, while we observed significant differences in immune cell infiltration and immune checkpoint expression between the two risk groups, the precise molecular mechanisms by which TMGs modulate the TME or contribute to immune evasion remain unclear. Future investigations should integrate single-cell transcriptomics, multi-omics approaches, and functional experiments to systematically explore the immunoregulatory roles of key TMGs and their potential as targets for combination immunotherapy. Lastly, our *in vitro* validation primarily focused on expression profiling, with limited functional characterization. Therefore, more comprehensive studies involving gene knockout or overexpression, as well as animal models, are necessary to strengthen the mechanistic evidence supporting our findings.

## Conclusion

This study is the first to systematically identify TMGs closely linked to the prognosis of CRC at the whole-genome level and to construct a CRC prognostic risk model consisting of seven key TMGs (CDC25C, CXCL1, RTL8C, FABP4, ITLN1, MUC12, and ERI1). The model demonstrated strong predictive ability across multiple independent cohorts and was also effective in identifying differences in the immune microenvironment and drug sensitivity of CRC patients. Our findings suggest that TMGs influence clinical outcomes in CRC patients by modulating tumor immune escape mechanisms. Combined with *in vitro* experiments, the expression of these key genes was found to be closely related to the invasive ability of CRC cells, further enhancing the biological relevance of the model. This study innovatively integrates the telomere maintenance mechanism with the potential for immunotherapy, providing candidate targets and a novel theoretical foundation for the management and development of targeted therapies for CRC.

## Supplemental data

**Table S1:** Primer sequences used in the study

**Gene**	**Accession no.**	**Primers (5′-3′)**	
		**Forward**	**Reverse**
*CDC25C*	NM_001790	AAGGCGGCTACAGAGACTTCTT	AGAGTTGGCTGGCTTGTGAGA
*CXCL1*	NM_001511	TGCTGCTCCTGCTCCTGGTA	GCTTTCCGCCCATTCTTGAGTG
*RTL8C*	NM_001078171	AAGCGAGGAGCAGCGATGGA	TGTGAGGCGGGTGATGAGGAA
*FABP4*	NM_001442	TGCAGCTTCCTTCTCACCTTGA	TGACGCATTCCACCACCAGTT
*ITLN1*	NM_017625	AACGCCTTGTGTGCTGGAATGA	ATCTCACGGCTGCTGCTGTAAC
*MUC12*	NM_001164462	CCTCAACTCACACGACGCCTTC	TGCTGCTGTAGACGGTGGTAGA
*ERI1*	NM_153332	ATCCTCTTGCCTCAGCCTCCT	TTCAAGACCAGCCTGACCAACA
*GAPDH*	NM_002046	GTCTCCTCTGACTTCAACAGCG	ACCACCCTGTTGCTGTAGCCAA

## Data Availability

The datasets generated and/or analyzed during the current study are available in the [GSE17537] repository, https://www.ncbi.nlm.nih.gov/geo/query/acc.cgi?acc=GSE17537.
